# Emerging Roles of Complement in Psychiatric Disorders

**DOI:** 10.3389/fpsyt.2019.00573

**Published:** 2019-08-21

**Authors:** Mélanie Druart, Corentin Le Magueresse

**Affiliations:** ^1^INSERM UMR-S 1270, Paris, France; ^2^Science and Engineering Faculty, Sorbonne Université, Paris, France; ^3^Institut du Fer à Moulin, Paris, France

**Keywords:** brain development, schizophrenia, synapse elimination, synaptic pruning, microglia

## Abstract

The complement system consists of more than 30 proteins that have long been known to participate to the immune defence against pathogens and to the removal of damaged cells. Their role, however, extends beyond immunity and clearance of altered “self” components in the periphery. In particular, complement proteins can be induced by all cell types in the brain. Recent discoveries highlight the role of complement in normal and pathological brain development. Specifically, the complement system mediates synaptic pruning, a developmental process whereby supernumerary synapses are eliminated in the immature brain. The complement system has been implicated in pathological synapse elimination in schizophrenia, West Nile virus infection, and lupus, all of which are associated with psychiatric manifestations. Complement also contributes to synapse loss in neurodegenerative conditions. This review provides a brief overview of the well-studied role of complement molecules in immunity. The contribution of complement to embryonic and adult neurogenesis, neuronal migration, and developmental synaptic elimination in the normal brain is reviewed. We discuss the role of complement in synapse loss in psychiatric and neurological diseases and evaluate the therapeutic potential of complement-targeting drugs for brain disorders.

## Introduction

The link between immunogenetics, inflammation, and several major psychiatric disorders such as Schizophrenia (SZ), Bipolar Disorder (BD), and Autism Spectrum Disorder (ASD) is now well substantiated (1–5). The molecular and cellular mechanisms that mediate immunity-related neurodevelopmental alterations in psychiatric diseases are gradually coming to light, and the complement system appears to be a key player in these complex processes. The complement system is an ensemble of proteins that collectively participate to host defense against infections by opsonizing antigens, promoting inflammation, and lysing pathogens, and has been well characterized in the periphery. Unexpectedly, complement was shown to control synaptic pruning, a development process whereby supernumerary synapses are eliminated during the course of normal brain maturation (6). Recently, the complement system has received much attention in the field of psychiatry after Sekar et al. demonstrated that distinct genetic variants of C4, a gene encoding a protein of the classical complement pathway, predispose to SZ (7). This landmark study provided a solid basis for establishing a causal relationship between complement-mediated synaptic pruning and cortical thinning frequently associated with SZ. Furthermore, complement-mediated synapse loss was also implicated in Lupus and West Nile virus infection, two immune disorders that can induce psychosis and cognitive impairment, respectively. In this review, we briefly summarize the known functions of the complement system in the periphery and highlight its newly discovered roles in brain maturation and in psychiatric disorders.

### The Three Complement Cascades in Innate and Acquired Immunity

The complement system consists of more than 30 proteins present in the blood plasma or on the cell surface. These proteins are mainly produced by the liver, although multiple cell types in different organs, including the brain, express and secrete complement molecules (8). The complement system has been extensively studied, and numerous reviews cover complement pathways in depth (9–12). Complement molecules have three major functions in the immune system: i) opsonization of “foreign” molecules, bacteria, or damaged cells, i.e., promotion of their phagocytosis by neutrophils or monocytes, ii) increase of the inflammatory response via short peptides called anaphylatoxins (C3a, C4a, and C5a) resulting from enzymatic cleavage of complement proteins, and iii) lysis of pathogenic micro-organisms via the formation of a pore in the lipid membrane.

**Figure 1:**
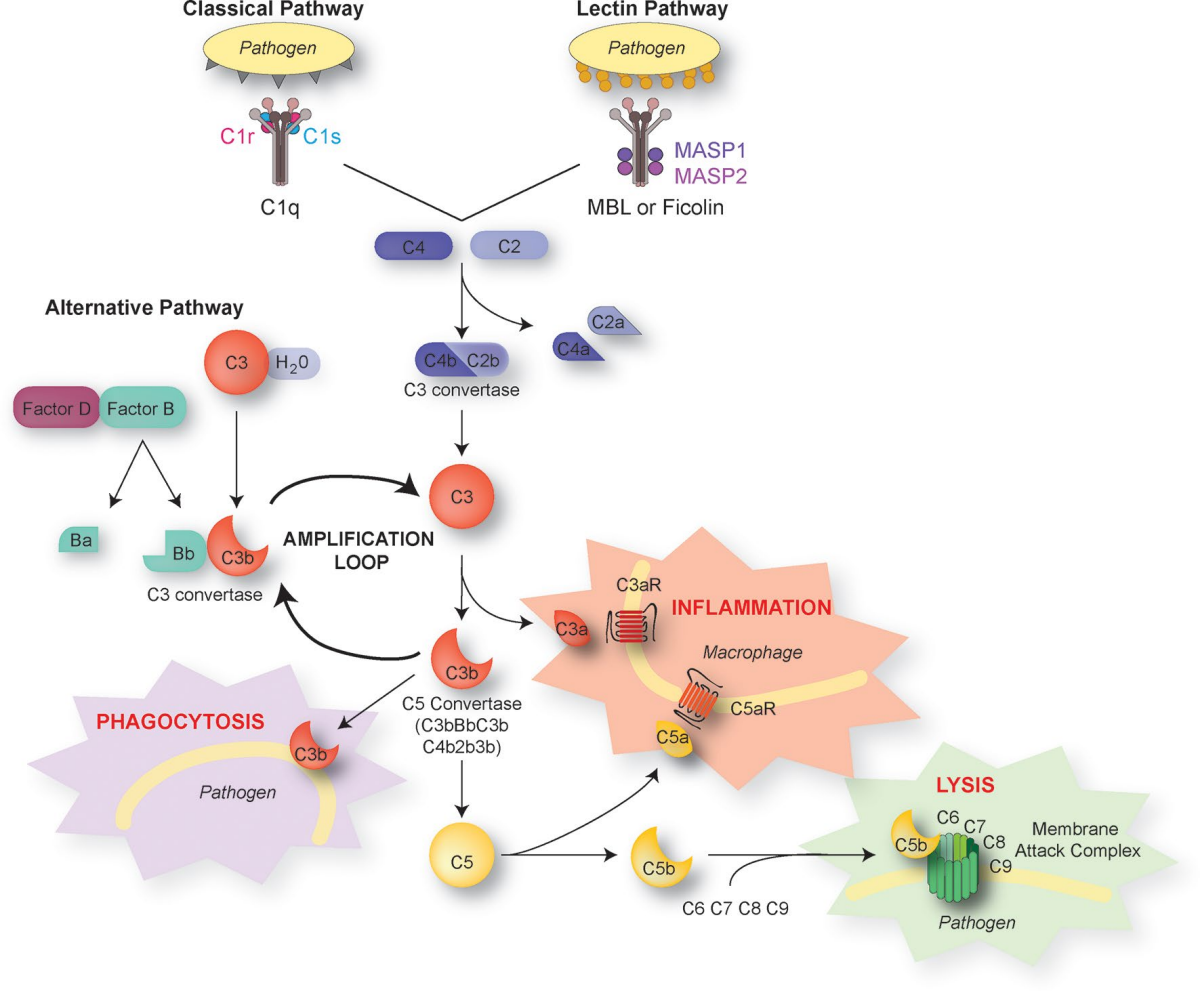
The complement pathways. There are three distinct activation pathways in the complement system: the classical and lectin pathway that are activated by pathogens or damaged cells, and the alternative pathway that is activated by the spontaneous hydrolysis of C3. All lead to the sequential recruitment of complement components to form a C3 convertase (C4bC2b or C3bBb). The C3 convertase cleaves C3 into C3a and C3b. The C3b fragment binds to the surface of antigens, targeting them for opsonization (phagocytosis). C3b also induces a positive-feedback loop (“amplification loop”) leading to the generation of additional C3 convertase. C3b can also be recruited to become part of the C5 convertase, which cleaves C5 into C5a and C5b. The short C3a and C5a fragments, also known as anaphylatoxins, foster immune responses and inflammation. C5b initiates the assembly of C6, 7, 8, and 9 into the membrane attack complex, which forms a membrane pore resulting in the lysis of the pathogen.

Complement proteins are soluble and circulate in the blood in an inactive form. In response to an activating mechanism, some complement proteins are transformed into proteases and cleave other specific complement members, initiating amplification cascades (**Figure 1**). Complement activation results from the activation of one or several of three distinct pathways: the classical pathway, the lectin pathway, and the alternative pathway. The three pathways converge to cause cleavage of C3, which is the most abundant complement protein, resulting in the formation of the anaphylatoxin C3a and the C3b fragment. Factor I, in the presence of its co-factor and C3b receptor CR1, cleaves C3b into iC3b and a small peptide of 17 amino-acids (C3f). The resulting conformational rearrangement of iC3b generates binding surfaces for the interaction with complement receptors CR2 (CD21), CR3 (CD11b/CD18), and CR4 (CD11c/CD18) located on leukocytes, which down-regulates inflammation and increases B cell sensitivity, thus forming a link between the innate and adaptive immune systems (13, 14). The C3b fragment can also directly label antigens for opsonization or bind to other complement peptides to form the C5 convertase. The resulting cleavage of C5 is the beginning of the terminal pathway of the complement: C5 is cleaved into C5a, the most potent anaphylatoxin, and C5b. The latter forms a complex with C6 and C7, which then binds with C8. The resulting change in C8 conformation allows the insertion of an alpha chain and anchoring in the target membrane. The combination of the C5b678 complex with one to 18 C9 molecules, termed membrane attack complex, forms a pore that lyses the target pathogen.

#### The Classical Pathway

The classical pathway is activated by the C1 complex, composed of the C1q protein recognizing antibodies bound to their antigen, associated with the C1r and C1s proenzymes. Through C1q, the complex can also recognize non-immunoglobulin activators, particularly surface proteins of bacteria and viruses. The binding of C1q to its target results in a conformational change of C1q which activates C1r, which in turn cleaves and activates the two C1s molecules of the C1 complex to form the activated C1s serine protease. Activated C1s cleaves C4 and C2, whose cleavage products combine to form the C3 convertase C4b2b which activates C3.

#### The Lectin Pathway

The lectin pathway is activated by the binding of pattern recognition receptors such as mannose-binding lectins, ficolins, or collectin 11 to specifically arranged carbohydrates present on damaged cell surfaces or on invading pathogens. This results in the activation of associated serine proteinases (MASP1/2) and cleavage of C4 and C2. The formation of the C3 convertase C4b2b then cleaves C3 as in the classical pathway.

#### The Alternative Pathway

Unlike the other two pathways, the alternative pathway is not initiated by binding of complement molecules to antigens or antibodies. Spontaneous hydrolysis of C3 results in the cleavage of an intramolecular thioester bond and leads to the formation of reactive molecules C3(H_2_O) and C3b. When C3(H_2_O) or C3b binds to positively charged surfaces present in microorganisms, it reacts with Factor B in the presence of Factor D to form a C3 convertase (C3(H_2_O)Bb or C3bBb) that is strongly stabilized by properdin (Factor P). The convertase then generates additional C3b molecules, initiating a positive feedback loop amplification.

### Complement Proteins in Normal Brain Development

#### Expression of Complement Components in the Brain

Studies conducted since the 1990s have shown extrahepatic production of complement proteins by several organs and cell types (8) and, in particular, by neurons and glial cells (15). This local production in the CNS is all the more important as the brain-blood barrier prevents circulating macromolecules, including complements proteins, to penetrate into the brain tissue (16).

In the past two decades, expression of complement components has been documented in primary cultures of astrocytes, microglia, oligodendrocytes, and neurons (17–20). More recent studies have shown that neural cells express complement molecules throughout embryonic and postnatal development in rodent and human brain tissue (**Table 1**) (25, 33–35). Importantly, complement components are released into the extracellular space, suggesting that a given brain cell type can participate to complement activation without expressing the full range of complement molecules (7).

**Table 1:** Complement molecules expressed by brain cells.

	Astrocytes	References	Microglia	References	Oligodendrocytes	References	Neurons	References
**Classical pathway**	C1q, C1r, C1s, C2, C3, C4	Barnum et al. (21) Veerhuis et al. (19)	C1q, C1r, C1s, C2, C3, C4	Walker et al. (18) Haga et al. (22) Veerhuis et al. (19)	C1q, C1r, C1s, C2, C3, C4	Hosokawa et al. (23) Gasque and Morgan (24)	C1q, C1r, C1s, C2, C3, C4	Shen et al. (25) Thomas et al. (20)
**Alternative pathway**	C3, Factors B, D, I, H	Barnum et al. (21) Gordon et al. (26)	C3	Veerhuis et al. (19)	C3, Factor H	Hosokawa et al. (23) Gasque and Morgan (24)	C3, Factors B and D	Thomas et al. (20)
**Lectin pathway**							(embryonic neurons) MASP1, MASP2	Gorelik et al. (27)
**Terminal pathway**	C5,C6,C7,C8,C9	Gasque et al. (28)			C5, C6, C7, C8, C9	Hosokawa et al. (23) Gasque and Morgan (24)	C5, C6, C7, C9	Shen et al. (25) Thomas et al. (20)
**Receptors**	CR1, CR2, C3aR, C5aR	Gasque and Morgan (24) Gasque et al. (29) Gasque et al. (30)	CR2, C3aR, C5aR	Gasque et al. (30) Davoust et al. (31)			C3aR, C5aR	Davoust et al. (31) Stahel et al. (32) Benard et al. (33)

Complement expression by neural cells was first demonstrated using immunoprecipitation in cultured astrocytes (17). This observation was compatible with the macrophagic function of astrocytes and their role in the brain’s immune system (36). Astrocytes express components of the classical pathway (C1q, C1r, C1s, C2, C3, C4), the alternative pathway (factors B, D, I, H, P), and the terminal pathway (C5-C9) (19, 21, 26, 28). They also express complement receptors CR1, CR2, C3aR, and C5aR (29, 30). These initial studies relied on cultured cell lines. In the mouse brain, astrocytes are the main source of C3 (37). Like astrocytes, microglia are considered part of the immune system of the brain because of their ability to secrete pro-inflammatory factors such as chemokines and cytokines and for their macrophagic function (38, 39). Microglial cells express classical pathway components (C1q, C1r, C1s, C2, C3, C4) and complement receptors C1qR, CR2, C3aR, and C5aR (19, 22, 40–43). Microglial cells have been shown to be the dominant source of C1q in the mouse brain (44). Oligodendrocytes also express members of the classical pathway (C1q, C2, C3, C4), factor H, as well components of the terminal pathway (C5-C9) (23, 24).

Finally, neurons also express complement molecules. In culture, they can produce a complete complement system (20). mRNA coding for C1q, C2, C3, C4, and terminal complement components (C5-C9) was also detected in human neurons using *in situ* hybridization in *post mortem* brain tissue (25). Immunohistochemical experiments using human cortical neurons in culture showed the presence of the C4 protein in neurons and in the extracellular medium, confirming that neurons express and release C4 (7). Altogether, these results suggest that both neurons and glial cells express complement components and their receptors. Given the pro-inflammatory role of anaphylatoxins C3a and C5a, the expression of their receptors by glial cells was not surprising. In contrast, expression of these receptors in neurons was more unexpected. Yet, neuronal expression of C3aR and C5aR at a low level has been shown in the cortex, cerebellum, and hippocampus in the adult mouse brain (31, 32, 45).

Complement expression varies according to the brain’s inflammatory status. In response to infection or inflammation such as in bacterial meningitis, elevated levels of complement proteins are detected in the cerebro-spinal fluid (46). Regulation of complement receptor expression was shown in an ischemic brain model where C3aR and C5aR are more expressed in both neurons and glial cells following blood vessel occlusion (47). Interestingly, a cell type-specific upregulation of complement expression has been observed in a model of transient ischemia where C1q expression increases specifically in microglia but not in neurons (48). Expression of complement components and receptors also varies during the course of brain development (33). For example in the mouse hippocampus, C3 expression is much lower at postnatal day 30 (P30) than at P2 (35). In the rat cerebellum, C3aR and C5aR expression in granule cells peaks around postnatal day 12 (33). This fine developmental regulation of complement expression has led to the study of its role in brain maturation.

#### Embryogenesis and Neuronal Proliferation

A study conducted in *Xenopus larves* highlighted the expression of complement components already during gastrula/early neurula stage. In particular, properdin, C1qA, C3, and C9 are expressed in the neural plate and in neural precursors, while C1qR and C6 are expressed at the periphery of the neural plate, in the presumptive neural crest (49). Based on the observation that there is a chronological and tissue specification of complement expression, the hypothesis of the complement’s involvement in developmental processes independently of inflammation has been put forward. In mammals, C5 and C5aR are also expressed early in development. C5 and C5aR are located in neuroepithelium in mice in the early stages of neurulation and also in human neuroepithelium. C5aR-deficient mice do not display overt congenital anomalies but have more neural tube malformations than wild-type controls after maternal folate deficiency (50). These observations suggest a degree of functional redundancy of developmentally expressed complement proteins, with the role of C5aR in neurulation only becoming apparent under conditions of environmental stress.

The C5a-C5aR axis is involved in neurogenesis, but only at embryonic stages of development. A recent study showed that C5aR activation increases neural recursor cell (NPC) proliferation *in vivo* in the embryonic ventricular zone through PKCζ/ERK signaling and that conversely, pharmacological blockade of C5aR decreases proliferation (51). In contrast, mice lacking C5aR do not show altered adult neurogenesis (52). Similarly, C5aR antagonists do not alter neural NPC proliferation within the external granular layer of the early postnatal rat cerebellum. However, C5aR agonists promote the proliferation of NPCs in the granular layer at the same developmental stage, suggesting that C5aR is expressed but not activated (53). The effect of C5aR activation at embryonic, but not postnatal stages, can be explained by the developmental time course of C5a expression. Indeed, the concentration of C5a is higher in embryonic than in adult cerebro-spinal fluid, suggesting that neuroepithelium secretes high quantities of C5a to promote NPC proliferation (51). The transient disruption of C5a-C5aR signaling during embryonic development alters adult cerebral organization and causes behavioral deficits (51), highlighting the crucial role of this pathway for the establishment of functional neuronal circuits. Interestingly, *Serping1*, a gene encoding a C1 inhibitor known to block the initiation of the classical and lectin pathways, negatively regulates neural proliferation in the embryonic ventricular zone by decreasing C5aR activation (54). Thus, C5aR activation must be precisely balanced for adequate NPC proliferation in the embryonic ventricular zone.

Complement molecules other than C5a are also involved in the control of adult neurogenesis. Thus, complement receptor CR2 is expressed by neural progenitors in the adult dentate gyrus (DG) and its activation by C3d or interferon-alpha reduces NPC proliferation and decreases the formation of new neurons in the adult hippocampus. Conversely, Cr2^−/−^ mice exhibit increased neurogenesis in the adult DG (55). C3a is also implicated in normal and ischemia-induced adult neurogenesis. C3aR is expressed by neural progenitor cells (NPC) in adult mice. Basal adult neurogenesis is decreased both in C3-deficient mice and in mice lacking C3aR. Furthermore, C3-deficient mice have impaired ischemia-induced neurogenesis in the subventricular zone, the main source of neural progenitor cells in the adult mouse brain (56).

#### Neuronal Migration

The complement system also plays a role in neuronal migration. During brain development, cells can migrate in a coordinated way in the same direction, a process termed collective migration that requires chemoattraction between cells. In neural ridge cells from Xenopus laevis and zebrafish, collective migration is dependent on the C3a fragment and its receptor C3aR. Disruption of the interaction between the ligand and its receptor prevents proper cellular migration and causes neuronal dispersion (57). A possibly related mechanism has been reported in adult brain-derived NPCs in which C3a and SDF-1 induce ERK phosphorylation, which in turn causes differentiation and neuronal migration in vitro (58).

Furthermore, knockdown of C1q inhibitor-encoding *Serping 1* impairs radial migration. Interestingly, *Serping 1* affects both cell autonomous and non-cell autonomous radial migration in mouse embryos, indicating that C1q inhibitor is secreted and influences neighboring neurons. This effect can be rescued C3aR agonists, demonstrating that C3aR signaling is required for the proper migration of cortical plate neurons (54). Further experiments have shown that the lectin pathway controls neuronal migration in the developing neocortex. Indeed, in mouse embryos knocking down C3, but also MASP1 or MASP2, which are critically involved in the lectin pathway, impairs migration of neuroblasts derived from the ventricular zone. This effect can be reversed by co-electroporating molecular mimics of C3 cleavage products, suggesting that the activity of the complement pathway, and not only the presence of the C3 protein, is required for proper migration of neuroblasts in the developing cortex (27).

#### Synaptic Pruning

In the past decade, a key role of the complement in the postnatal maturation of brain circuits has been uncovered. At birth, the mammalian brain is characterized by an excess of synaptic connections. Over the course of postnatal development, extra synapses are eliminated to establish functional mature neuronal networks (59, 60). This process called synaptic pruning is activity-dependent and has been documented in different regions such as the cortex (61, 62), cerebellum (63), retinogeniculate system (59), and neuromuscular junction (64).

**Figure 2:**
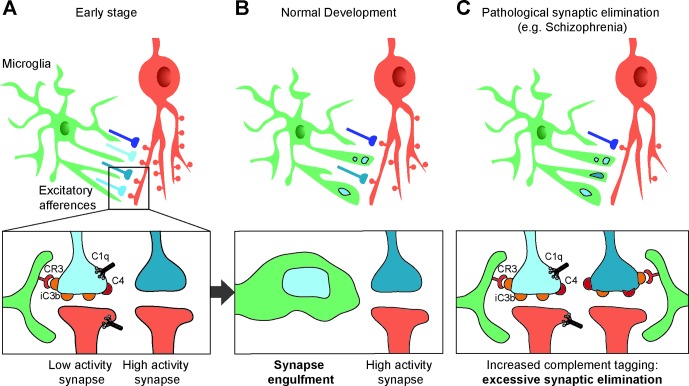
Synapses elimination by microglia in normal and pathologic development. **(A, B)** During the course of postnatal development, supernumerary synapses are eliminated by microglia (upper panels). Complement components C1q, C4, and the C3 fragment iC3b tag inactive synapses. Microglial cells bind iC3b through their CR3 receptors and partially phagocyte tagged synapses, resulting in selective synapse elimination. High-activity synapses appear to be protected from synaptic pruning by an activity-dependent signal (bottom panels). **(C)** Synaptic pruning hypothesis in schizophrenia: high C4A expression in the schizophrenic human brain and the associated decrease in synapse density suggest increased complement activity resulting in an excess of iC3b-marked synapses and faulty synapse elimination **(C)**.

The role of complement in synaptic pruning has been first demonstrated in the retinogeniculate system (**Figure 2A, B**). Early in development, retinal ganglion cells (RGC) project exuberant axons onto neurons in the dorsal lateral geniculate nucleus (dLGN) of the thalamus. During postnatal development, retinogeniculate synapses are eliminated to ensure a good segregation between ipsi and contralateral inputs to dLGN neurons. Activity-dependent pruning that occurs in the first postnatal week in mice is necessary to provide a functional binocular vision in adults (65, 66). Using array tomography on thin brain sections, Stevens and colleagues showed that C1q and C3 are abundantly expressed and colocalize with excitatory synapses in the dLGN at P5 but not at P30. Consistent with a role of complement in synaptic pruning, C1q-, C3-, and C4-deficient mice display higher densities of excitatory synapses in the dLGN and abnormalities in eye-specific segregation (6, 7). The colocalization between C3 and excitatory synapses suggests that C3 or its cleavage products act like tags of synapses to be eliminated by microglia. Indeed, if the interaction between iC3b and its receptor CR3 is blocked, synaptic pruning is impaired (67). CR3 is selectively expressed by microglial cells during postnatal development and anterograde tracer injection in the retina showed that presynaptic elements are engulfed by microglial cells in the developing dLGN, indicating that microglial cells are key players in complement-mediated synaptic pruning (67). C1q and C4 also colocalize at synapses in the developing dLGN (6, 7). However, it is unclear whether they directly participate in microglia-dependent synaptic elimination by tagging synapses, or whether they indirectly promote synapse elimination by allowing the cleavage of C3 and the generation of iC3b.

Consistent with numerous studies highlighting that synaptic pruning is activity-dependent, microglia-mediated engulfment of RGC inputs is regulated by the activity of presynaptic neurons (67). This further suggests that inactive synapses are those that are tagged by complement molecules and targeted for microglia-mediated pruning, although the mechanisms underlying activity-dependent complement binding remain unknown.

The role of complement in synaptic pruning has also been demonstrated in other brain regions. Thus, the complement system contributes to pruning of axonal boutons in layer V pyramidal neurons of the somatosensory cortex (68). A role of microglia in synaptic pruning has also been demonstrated in the hippocampus (69, 70), suggesting that complement-dependent synaptic pruning may be widespread in the developing brain. A recent study shed light on microglia-neuron interactions in the developing hippocampus at the ultrastructural level, showing that presynaptic structures in developing hippocampal neurons are not entirely phagocytosed by microglia but rather “trogocytosed,” a term originally used to describe membrane transfer in immune cells and later extended to refer to partial phagocytosis (71). Of note, microglia-independent processes also participate to synaptic pruning. Thus, astrocytes contribute to synapse elimination in the developing and adult dLGN through the MEGF10 and MERTK phagocytic pathways (72). The contribution of complement to astrocyte-mediated pruning is unclear, but the identification of MEGF10 as an astrocytic receptor for C1q in the developing mouse cerebellum (73) raises the possibility that astrocytes recognize C1q-tagged synapses for elimination.

#### C1q Family

Interestingly, proteins that have homology with C1q but are not involved in the leptin, classical, or alternative pathways are expressed in the brain and participate in synapse formation, maintenance, and function. These proteins of the C1q family include precerebellin (Cbln1), which is released by cerebellar granule cells. Cbln1 promotes synapse formation between granule cell axons (parallel fibers) and Purkinje cells through binding to glutamate receptor delta 2 (GluRD2) on postsynaptic site and to Neurexin on presynaptic sites. C1q-like (C1ql) proteins, another subtype of C1q family proteins, also regulate synapse formation and function in the cerebellum and in the forebrain. For example, in the cerebellum, C1ql1 controls the formation and maintenance of synapses between axons of the inferior olivary nucleus (climbing fibers) and Purkinje cells through binding to the postsynaptic receptor Bai3 (74–76). Two other C1ql proteins, C1ql2 and C1ql3, are strongly expressed by dentate granule cells in the hippocampus and recruit postsynaptic kainate receptors at mossy fiber-CA3 synapses (77). However, proteins of the C1q family other than C1q do not participate in the molecular cascades resulting in the cleavage of C3, and hence are not part of the complement system per se.

### The Complement System in Psychiatric Diseases

#### Schizophrenia

Compelling evidence links the complement system and SZ (**Figure 2C**). Following the demonstration that synaptic density in neocortex peaks in early childhood, then decreases before reaching a plateau in adolescence (61), Feinberg proposed that SZ may result from faulty synapse elimination in the postnatal period based in particular on age at onset (78). Several observations corroborated Feinberg’s hypothesis. First, spine density is decreased in upper cortical layers in SZ patients (79, 80). Second, MRI studies revealed that cortical thinning that takes place during normal postnatal development in humans is exacerbated in SZ patients, possibly reflecting an excess of pruning (81, 82). Given the now well-established role of complement in developmental synaptic pruning, it is not surprising, in retrospect, that alterations in complement genetics or complement expression have been associated with SZ. Early studies of the complement system in SZ showed altered expression or activity of complement molecules in SZ, although the results were inconsistent. In particular, increased hemolytic activity of the complement Cl, C2, C4 components in SZ had been reported by several studies (83, 84) [for a thorough review, see (85)]. However, the small scale of these studies prevented firm conclusions. A more robust association between the complement system and SZ has been made possible by large-scale genome-wide association studies of SZ. A significant association was found on chromosome 8 near *CSMD1* (86, 87), which encodes a complement-regulating protein (88). CSMD1-deficient mice display behaviors that are reminiscent of SZ-associated blunted emotional responses, anxiety, and depression (89). Furthermore, several GWAS have indicated that the Major Histocompatibility Complex (MHC) genomic region on chromosome 6 is implicated in SZ. Given that the strongest association within the MHC lies near the genomic region encoding complement component 4 (*C4*), Sekar et al. explored in detail the C4 complement cluster of the MHC in SZ patients and control individuals. They demonstrated a strong relationship between the C4 region and SZ (7). The tandemly arranged C4A and C4B genes in the MHC class III region are polymorphic in terms of copy number variation (CNV) and structure. They have over 95% sequence homology but encode functionally distinct proteins with different molecular targets. Further complexity stems from the fact that both C4A and C4B can be found in either “long” or “short” forms depending on the presence of human endogenous retroviral (HERV) indels (6.5 kb in size) in the intron 9 of the C4 genes. High copy number and the presence of HERV elements increase the mRNA levels of C4A and C4B. Importantly, the presence of multiple copies of C4A and of long forms of C4A was shown to increase the risk for SZ, suggesting that higher C4A expression predisposes to SZ. Indeed, in a separate set of experiments Sekar et al. used postmortem expression analysis to demonstrate that C4A is significantly more expressed in the brain of SZ patients than in the brain of control individuals (7). A subsequent brain imaging study using phosphorus magnetic resonance spectroscopy directly confirmed the association between C4A gene repeats and neuropil contraction in two cohorts of SZ patients. Increased neuropil contraction was observed in the prefrontal and parietal regions among adult-onset schizophrenia patients with high C4A gene copy numbers, whereas adolescent-onset SZ patients showed increased neuropil contraction in the prefrontal cortex and thalamus (90).

#### Immune Disorders With Psychiatric Manifestations

Systemic lupus erythematosus (SLE) is a relatively rare chronic autoimmune disease. In most patients with SLE, anti-nuclear autoantibodies cause chronic inflammation which damages tissues, leading to a variety of symptoms. Lupus can affect many organs such as the kidneys, heart, lungs, blood vessels, and brain. Neuropsychiatric manifestations are present in two-thirds of the patients with SLE and include anxiety, depression, and psychosis, the latter being present in about 5% of patients (91). Gray matter atrophy was reported in SLE patients (92), which may be an indicator of synapse loss. In the 564Igi mouse model of SLE, a B-cell-receptor insertion model with known autoantibody specificity, behavioral phenotypes including anxiety, cognitive alterations, and social deficits correlate with synapse loss and the presence of reactive microglia. Treating 564Igi mice with an antibody against type I interferon receptor prevented reactive microglia, synapse loss, and behavioral phenotypes, suggesting that microglia-dependent synaptic elimination is involved in the behavioral alterations in SLE (93). Since the classical complement pathway is involved in microglia-dependent synaptic pruning in the healthy brain, it has been proposed that the complement system may directly or indirectly stimulate the type I interferon pathway to promote synapse engulfment and elimination by microglia in SLE (94).

The complement system has also been implicated in neuroinvasive infection with West Nile Virus. Cognitive decline including memory dysfunction is present in at least 50% of patients that survive following West Nile virus infection (95, 96). Using a mouse model of West Nile virus neuroinvasive infection, Vasek et al. showed that several complement proteins are significantly upregulated in the hippocampus, including C1q, C2, C3, and C4b (97). West Nile virus infection was accompanied by synaptic terminal elimination, which was blocked in C3-deficient mice and C3aR-deficient mice. Surprisingly, virus-induced synaptic elimination was still present in CR3-deficient mice, in which developmental synaptic pruning is strongly decreased (67). These results suggest that several distinct complement-dependent mechanisms may promote pathological pruning.

#### Neurodegenerative Disorders

While this review’s emphasis is on psychiatric disorders, it is noteworthy that the complement has also been implicated in neuron and synapse loss in neurodegenerative disorders. Increased expression of the complement has been noted in the brain following injury and neurodegeneration (98, 99). In Alzheimer’s disease (AD), the accumulation of extracellular Amyloid-beta (Aβ) peptides contributes to pathogenesis (100). Aggregated Aβ binds to C1q and C3b/iC3b and activates both the classical and alternative complement pathways in vitro (101, 102). Transcriptome studies showed that complement gene expression is increased in the disease, indicating activation of the complement system (103). Furthermore, several studies have demonstrated that the membrane attack complex colocalizes with amyloid plaques and tangle-bearing neurons, suggesting that complement activation in AD contributes to neurotoxicity (104–107). Indeed, C1q-deficient mouse models of AD display decreased levels of activated glia surrounding amyloid plaques, and slower decline of synaptic markers (108). Aged C3-deficient mice also show reduced age-dependent synapse and neuronal loss in hippocampal CA3, together with enhanced long-term potentiation and cognition (35). A recent study investigated in detail the role of the complement in synapse loss in the familial AD-mutant human amyloid precursor protein transgenic mouse model. In this model, C1q expression increases and C1q localizes to synapses before amyloid plaque deposition. Moreover, C1q-deficient mice are protected from Aβ-dependent synapse loss. These data suggested that the developmental pruning pathway is re-activated at the preplaque in AD, prompting the authors to test whether Aβ-dependent synapse loss is present in mice lacking CR3, a complement receptor only expressed by microglia in the brain. Synapse loss and microglial engulfment are prevented in CR3 KO mice (109). Another study has uncovered the contribution of C3aR, another complement receptor mainly expressed by microglia. Expression of C3 and C3aR is positively correlated with cognitive decline in human AD brains. In the PS19 mouse model, which is used to study neurofibrillary tangles in neurodegenerative taupathies like AD, a significant increase in C3 and C3aR expression correlates with synapse loss and microglial activation. Crossing PS19 mice with C3aR KO mice attenuates both synaptic impairment and microglial activation (110). Altogether, these results demonstrate the role of microglia and complement axis C3/CR3 and C3/C3aR in pathological synapse elimination. While research on complement activation in the AD brain has focused primarily on the classical complement pathway, it should be noted that mRNA for a critical alternative pathway component, Factor B, is present in the cortex of AD patients and that split products of Factor B, Bb and Ba, are significantly increased, indicating alternative pathway activation (111).

Pathological complement-mediated synapse loss is involved not only in AD but also in other neurogenerative disorders. In a progranulin-deficient mouse model of frontotemporal dementia, increased complement production and synaptic pruning activity by microglia preferentially eliminate inhibitory synapses in the ventral thalamus, which is prevented in progranulin-deficient C1q KO mice (112). C1q upregulation and C1q-dependent synapse loss were also observed in a mouse model of glaucoma, a neurodegenerative disease characterized by the progressive dysfunction and loss of retinal ganglion cells (113).

Complement molecules also participate indirectly in synapse loss and neurodegeneration in disorders such Alzheimer’s, Huntington’s, Parkinson’s, Amyotrophic lateral sclerosis, and Multiple Sclerosis by inducing reactive astrocytes. Upon microglial activation, C1q is released by microglial cells together with Il-1α and TNFα. The combined action of these three factors is sufficient to convert resting astrocytes into reactive “A1” astrocytes with impaired ability to promote synapse formation, decreased phagocytic capacity, and neurotoxicity (114).

It is remarkable that complement molecules participate to synapse loss in animal models of several distinct neurodegenerative disorders. While the neurotoxic effect of the complement may be less relevant for psychiatric disorders than for neurodegenerative disorders, similar complement-dependent mechanisms appear to induce synapse loss in both types of diseases (**Table 2**). Deciphering the underlying mechanisms in animal models of neurological disorders will help understand complement-dependent synaptic alterations in neurodevelopmental psychiatric disorders.

**Table 2:** Complement pathways involved in selected brain disorders.

	Neurodevelopmental disorder		Neuro-immune disorder	Neurodegenerative disorder	
	Schizophrenia		West Nile Virus	Alzheimer’s disease	
**Complement pathway**	Classical	Lectin	Classical	Classical	Alternative
**Putative complement activation**	Increased expression of C4A	Unknown	C1q binds to WNV antigen-positive neurons	C1q binds to Aβ	C3b/iC3b binds to Aβ
**Changes in complement components**	Increased C4 mRNA expression (human brain)	Increased MBL/MASP-2 complex activation (human serum)	Increased C1q, C2, C3 and C4b mRNA expression in the brain (mouse model)	Increased C1q, C3, C4, C9 mRNA expression (human brain) and increased C1q, C3, C3aR protein expression in the brain (mouse models)	Increased expression of split products of Factor B (Ba and Bb) (human brain)
**Cellular mechanisms involved**	Excess of microglia-dependent synaptic pruning	Unknown	Excess of C3/C3aR-dependent synaptic pruning	Excess of microglia-dependent synaptic pruning through the C3/CR3 and C3/C3aR axis	Unknown
**References**	Sekar et al. (7)	Mayilyan et al. (84)	Vasek et al. (97)	Jiang et al. (101) Blalock et al. (103) Hong et al. (109) Litvinchuk et al. (110)	Bradt et al. (102) Strohmeyer et al. (111)

### Perspectives

#### Is the Complement System Involved in Bipolar Disorder and Autism Spectrum Disorder?

Collectively, the studies discussed above have identified the classical complement pathway as a key contributor to pathological synapse elimination in the context of SZ and other disorders with psychosis or cognitive dysfunction. While the main emphasis has been on SZ, other psychiatric disorders such as BD and ASD are also associated with immune activation/inflammation (2–5) and altered synaptic pruning. Increased cortical thinning during adolescence has been observed in BD patients (115) and may reflect excessive synaptic pruning. Conversely, ASD has been associated with decreased synaptic pruning. Thus, in postmortem brain tissue, dendritic spine density was found to be increased in the cortex of ASD patients (116), which appears to result from decreased synaptic pruning as another study confirmed these results and showed that spine density decreased by ∼45% in control subjects from childhood through adolescence, but only by ∼16% in ASD patients (117).

Given the well-established role of complement molecules in both immune processes and synaptic pruning, these observations raise the question of whether the complement system may be causally involved in BD or ASD but with a different phenotypic expression than in SZ. The answer to this intriguing question may be multifold. First, clinically distinct psychiatric disorders sometimes display partly overlapping symptoms, which may reflect shared cellular endophenotypes including complement-mediated alterations at the synaptic level. Second, synaptic dysfunctions in SZ, BD, and ASD may rely on distinct mechanisms. The MHC region, which includes the C2, C4, and Factor B complement genes, has been associated with BD in genome-wide association studies (118), although the link between complement expression and BP is not straightforward (119). In contrast, preliminary results from a genome-wide association study on about 6500 ASD patients, a number that was sufficient to associate the MHC region in SZ (118), failed to reveal an association between the MHC and ASD (120). These results suggest that the MHC, which includes complement genes, might contribute to synapse elimination in BD, but seems less likely to be involved in ASD. Other mechanisms controlling spine density, such as autophagy, may underlie connectivity defects in some forms of ASD (117). Third, immune activation during brain development interacts with a genetic program that varies from one individual to another. Thus, genetic predisposition leading to strong expression of *C4A* could interact with brain inflammation to induce excessive synaptic pruning in SZ and possibly BD, while other variants, or other predisposition genes, would promote a different consequence of inflammation in ASD.

#### Types of Synapses Subject to Complement-Mediated Elimination

It is unclear whether complement-mediated synapse elimination targets both glutamatergic synapses and other synapses, such as GABAergic synapses, or even sites of neuromodulator (e.g., dopamine, acetylcholine, serotonin, noradrenaline) release. Microglia- and complement-mediated pruning has been extensively studied in hippocampal and thalamic excitatory neurons that bear dendritic spines (6, 7, 67, 69, 71). Moreover, developmental synaptic pruning in the primate cortex as well as excessive synaptic pruning in the schizophrenic cortex have been mostly studied, at the cellular level, using spine density as a readout (61, 79). Does this mean that dendritic spines are the physical substrate for complement-mediated pruning? Accumulating evidence shows that complement-mediated synapse pruning involves the elimination of presynaptic elements, but not postsynaptic material, by microglia (67, 71), although microglial engulfment of postsynaptic structures has also been reported (121). Thus, the disappearance of dendritic spines may follow the elimination of presynaptic elements, but is unlikely to be directly caused by complement-mediated microglial trogocytosis. Is complement-mediated synaptic pruning limited to glutamatergic synapses on dendritic spines? In this case, glutamatergic afferences onto GABAergic interneurons, which are almost deprived of dendritic spines, should be spared by complement-mediated pruning. Conversely, medium spiny neurons in the striatum, which bear numerous spines, may be particularly prone to undergo elevated synaptic pruning. This is important in the context of SZ, since typical antipsychotics primarily exert their function by blocking dopamine D2 receptors which are strongly expressed in medium spiny neurons. Interestingly, there is evidence that hippocampal GABAergic synapses are not affected by genetic manipulations altering microglia-neuron communication, suggesting that the mechanisms of pruning are different at excitatory and inhibitory synapses (70). In line with these findings, early electron microscopy studies in the macaque cortex showed a decrease in asymmetric (glutamatergic) synapse density, but not in symmetric (mostly GABAergic) synapse density during postnatal development (122). Furthermore C1q-deficient mice, which display alterations of synaptic pruning at excitatory synapses, show no significant change in inhibitory connectivity to layer V cortical pyramidal neurons (68). These studies supported the concept that complement- and microglia-dependent pruning preferentially affects excitatory synapses during postnatal development. However, C1q-dependent pruning of inhibitory, but not excitatory, synapses was recently demonstrated in the thalamus of progranulin-deficient mice (112). Moreover, the density of excitatory synapses on parvalbumin interneurons is lower in postpubertal relative to prepubertal monkeys, suggesting some degree of pruning at excitatory synapses onto aspiny neurons, although the role of complement was not investigated (123). Thus, different types of synapses may undergo complement-dependent pruning. Further studies are now needed to evaluate normal and pathological synaptic pruning in different neuronal types, brain areas, and neurotransmitter systems. If specific types of synapses are predominantly targeted by complement-mediated elimination, it will be important to decipher the molecular mechanisms that underlie this selectivity.

#### Effect on the Brain of Complement Molecules Generated Outside the Brain

Do complement molecules from outside the brain influence the central nervous system? Since most complement proteins do not cross the blood-brain barrier (BBB), it is likely that only complement molecules generated locally by neurons and glial cells shape normal brain development and function. Moreover, in SZ and AD, changes in complement levels in the brain are associated with upregulated complement expression by brain cells (7, 34). Nevertheless, pathological activation of complement in the periphery, for example, following immune challenges, may indirectly contribute to inflammation and physio-pathological processes in the brain by i) increasing the production of cytokines that cross the BBB and ii) compromising the BBB, which allows complement molecules of the periphery to enter the brain. Thus, BBB disruption is present in patients with SLE and the elevated level of C3 in the patients’ cerebro-spinal fluid was suggested to be, at least in part, attributable to the transfer of C3 from the systemic circulation (124). On the contrary, complement activation in the periphery can protect against brain-damaging infections. For example, the complement system controls West Nile virus infection by inducing a protective antibody response (125).

#### The Complement System as a Target for Therapeutic Intervention in Psychiatric Disorders

The first anti-complement drug used in the clinic, eculizumab, is the humanized form of a C5-specific monoclonal antibody. As early as 2004, clinical trials demonstrated its efficacy in treating paroxysmal nocturnal haemoglobinuria (PNH), a heamatological disorder that involves complement-dependent intravascular hemolysis (126). Chronic eculizumab treatment substantially reduces mortality and improves quality of life for patients with PNH (127). After the US Food and Drug Administration (FDA) approval of eculizumab for PNH treatment in 2007, eculizumab also received FDA approval for the kidney disease atypical haemolytic uraemic syndrome in 2011. In addition, four C1 inhibitors (Berinert, Ruconest, Cetor, and Cinryze) are currently approved for the treatment of hereditary angioedema (128). These successes renewed interest in complement-targeted drug discovery. Given the role of complement in innate immunity, the use of complement-targeting drugs has raised concerns about potential adverse side-effects. Indeed, patients with complement deficiencies are more likely to suffer from serious infections (129, 130). The infectious risk decreases when patients with complement deficiencies reach adulthood, suggesting that complement therapeutics may be safer past adolescence (131). Moreover, the long-term clinical use of eculizumab has allowed to gather favourable safety data, which has contributed to enhance the interest for complement therapeutics. Thus, several anti-complement drugs are in advanced-stage clinical trials, and many more are in development (128, 132). These compounds target several distinct complement proteins, including C1q, C1s, C2, Factor B, Factor D, C3, C5, C5a, and C5aR. Few target the central nervous system, for the role of complement in brain disorders has been established relatively recently and because of specific requirements for brain-targeting drugs. In particular, crossing the blood-brain barrier requires small lipophilic molecules, or molecules that can access carrier-mediated transport systems within the blood-brain barrier. A few recently developed anti-complement molecules have been shown to be effective in crossing the blood-brain barrier (133). Preclinical studies support the use of anti-complement drugs to prevent synapse loss in brain trauma and neurological disorders, but these findings have yet to translate into the clinic (134). Nevertheless, these investigations may pave the way for complement-based therapies in psychiatric disorders.

## Conclusions

In conclusion, our review outlines recent discoveries that link the complement system and psychiatric disorders. In the brain, the role of complement appears fundamentally different from its role in innate immunity, although key elements of the signaling cascades well described in the immune system are preserved. Indeed, most complement molecules of the three complement pathways, including those that form the membrane attack complex, are expressed in the brain. The specificity of complement actions in the brain seems to result from the specificity of the neural mechanisms involved, such as synaptic pruning, and from the expression of molecular targets that may not exist outside the brain, such as those located at synapses. These complement-binding molecules remain to be fully identified.

Early studies into the antibacterial action of serum complement can be traced back to the early 1790s and were continued by generations of scientific luminaries including Ilya Metchnikoff and Jules Bordet, as discussed by Sim et al. in a recent historical review (135). It is striking that research on the complement is still a source of major discoveries today and will provide exciting insight into normal and pathological brain development, a research area that might have seemed unlikely to the pioneers of the 18^th^, 19^th^, and 20^th^ centuries.

## Author Contributions

MD and CLM jointly wrote the article.

## Funding

MD was the recipient of a PhD fellowship from the doctoral school “Brain-Cognition-Behavior” (ED3C), Sorbonne Université, Paris. This work was supported by a grant from the BioPsy Laboratory of Excellence to CLM.

## Conflict of Interest Statement

The authors declare that the research was conducted in the absence of any commercial or financial relationships that could be construed as a potential conflict of interest.
